# Degradation of OLED performance by exposure to UV irradiation

**DOI:** 10.1039/c9ra09730a

**Published:** 2019-12-23

**Authors:** Sun-Kap Kwon, Ji-Ho Baek, Hyun-Chul Choi, Seong Keun Kim, Raju Lampande, Ramchandra Pode, Jang Hyuk Kwon

**Affiliations:** Department of Information Display, Kyung Hee University Dongdaemun-gu Seoul 02447 South Korea jhkwon@khu.ac.kr; Department of Physics, Kyung Hee University Dongdaemun-gu Seoul 02447 South Korea rbpode@khu.ac.kr; LG Display Wollong-myeon/Paju-Si Gyeonggi-do 413-779 South Korea

## Abstract

Organic light-emitting diode (OLED) displays are highly susceptible to the harsh environmental conditions found outdoors, like exposure to direct sunlight as well as UV radiation and storage temperature, resulting in a loss of luminance and lifespan, pixel shrinkage, and permanent damage and/or malfunction of the panel. Here, we fabricated top emission OLEDs (TEOLEDs) using Yb : LiF (1 : 1, 2 nm)/Ag : Mg (10 : 1, 16 nm) and Mg : LiF (1 : 1, 2 nm)/Ag : Mg (10 : 1, 16 nm) cathode units and the performances of the devices were investigated by subjecting them to UV radiation. A fabricated red TEOLED (control device), employing a standard Mg : LiF (1 : 1, 2 nm) electron injection layer (EIL) and an Ag : Mg (16 nm) cathode, showed a rapid decrease in luminance and a fast increase in driving voltage at 10 mA cm^−2^ over time after UV irradiation for 300 h. However, a cathode unit comprising a Yb : LiF (1 : 1, 2 nm) EIL and an Ag : Mg (10 : 1, 16 nm) cathode showed no loss of luminance or increase in driving voltage at 10 mA cm^−2^ over time after UV irradiation for 300 h. Therefore, we investigated the changes occurring in both cathode units due to UV irradiation using the lift-out FIB-TEM technique and EDS mapping. With UV irradiation for 300 h, Ag atoms migrated toward the center of the cathode, Mg atoms migrated toward the CPL, and no Mg atoms were observed in the EIL area. In contrast, we observed (i) no substantial migration of Ag atoms and they were located at the center of the cathode, (ii) no migration of Mg atoms toward the CPL layer, and (iii) no movement of Yb atoms after UV irradiation. Furthermore, the UV irradiated red TEOLED with an Mg : LiF (1 : 1, 2 nm) EIL showed (i) deterioration in electron injection into the emissive layer (EML) and an increase in the EIL/metal interface resistance, and (ii) a remarkable shift of the *J*–*V* curve to the higher voltage side, while almost no such changes were observed in the TEOLD with a Yb : LiF (1 : 1, 2 nm) EIL. Also, an almost identical RGB pixel emitting area was noticed in the Yb : LiF (1 : 1, 2 nm) based devices after UV irradiation for 300 h. These results suggest that Yb could become a good candidate for the cathode unit, providing better device stability against harsh environmental conditions as well as excellent electron injection properties.

## Abstract 2

This paper reports the influence of UV radiation on the interface of a co-deposited EIL and Ag : Mg (10 : 1) cathode unit and pixel shrinkage.

## Graphical abstract

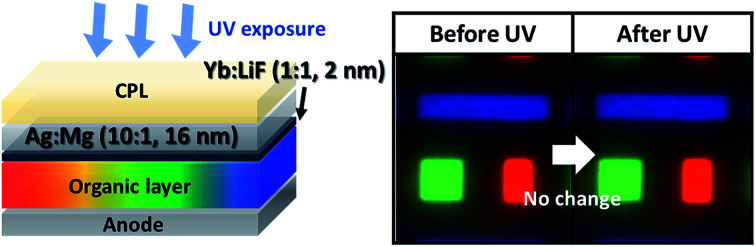

## Introduction

Organic light-emitting diode (OLED) devices have achieved commercial success for displays used in cell-phones, televisions, and other applications. Despite these achievements, harsh environmental factors outdoors cause serious concerns about operational tolerance. Such technical challenges facing OLED display technology need to be addressed to achieve wider acceptance. The environmental factors (solar radiation, humidity, oxygen, harsh operational temperatures outdoors), internal factors (OLED material degradation), aging of the devices (storage temperature), and contraction of various organic, inorganic, and/or metal cathode layers used in device fabrication cause a loss of luminance and a decrease in the lifespan of OLEDs, and also cause shrinkage of the active pixel area.^1^

Earlier, several studies observed a gradual decrease in the luminance, which could result either from the natural operation of the OLED (dark spot formation),^2^ or chemical degradation of different materials in the OLEDs or be caused by exposure to external radiation, especially to ultraviolet (UV) radiation.^3^ The interfacial damage at the electrode/active layer interfaces of the OLED devices has been found to be the cause of the photo-degradation of the luminance of OLEDs.^3^ Earlier, Heil *et al.* revealed that exposure to sunlight considerably decreases the electroluminescence (EL) intensity and the current of an OLED device, while the photoluminescence remains unaffected.^4^ Photo-degradation at the indium tin oxide (ITO)/polymer interface was reported to be the main cause for such effects, while the ITO and active polymer layers were insensitive to visible or near-ultraviolet light irradiation. In 2010, Wang *et al.* reported the influence of UV and 465 nm light illumination for different time durations on the current efficiency of blue-emitting devices and they revealed that the EL efficiency decreases gradually due to changes in the metal cathode (Mg : Ag) or at the cathode/organic interface, causing electron injection to become more difficult.^5^ Reese *et al.* showed significant degradation of the metal/organic interface in the devices under a constant illumination of solar radiation (10% duty cycle of 1 sun illumination, with a 1000 W Sunmaster metal halide lamp) for over 200 h.^6,7^

Recently, Askola *et al.* studied the photo-degradation of two commercially available white OLEDs using UV radiation (one device exposed to uniform UV A/B radiation and the other to spectrally resolved UV radiation from 273 nm to 365 nm).^8^ The UV exposure increased the luminance decay by at most a factor of eight compared to the natural aging observed from the same device. An effective way of addressing these issues, and improving the device efficiency and lifetime are vital if OLEDs are to achieve wide acceptance. Furthermore, a decrease in transmittance from 350 nm to 500 nm in pure silver (Ag) film was noticed due to an increase in the chemical reaction of Ag with absorbed oxygen molecules, whereas much less degradation or almost negligible degradation even after 17 h of UV radiation irradiation on Ag : Au (5.2%) alloy film (deposited by vacuum thermal evaporation) was reported by Ming Zhou *et al.*^9^ Considering this, the occurrence of some kind of modification in the composition of the metal cathode or at the electron injection layer (EIL)/metal cathode interface due to UV exposure seems to be one of the major causes of loss of intensity and performance degradation of OLED devices.

Normally, the standard Mg : Ag (10 : 1) cathode is widely used in OLED devices owing to the low work function of Mg that is also advantageous in electron injection and device stability. But, a cathode made of a higher Mg concentration has a great tendency to absorb light due to its high extinction coefficient. To address such an issue, thin-film cathodes containing a higher percentage of Ag have been suggested. However, the higher percentage of Ag may hinder the injection of electrons and lead to a higher driving voltage of the OLED device. To improve the electron injection and transmittance characteristics of a conventional Mg : Ag thin-film cathode in TEOLEDs, EIL materials such as LiF/aluminum (Al), Mg, Ag, ytterbium (Yb), rubidium (Rb), cesium (Cs), barium (Ba), Al, *etc.* have been suggested.^10^ The electrical and physical properties of Ag, Mg, and Yb are presented in Table 1 for a better comparison.^11^ Earlier, the use of co-deposited EIL systems, such as Yb : LiF and Mg : LiF, was reported for OLED devices with enhanced electron injection properties and improved device stability compared with standard EILs (LiF).^1,12,13^ Most of the earlier results about Yb metal usage are related to an improvement in the electron injection performance of the cathode unit.^14–16^ To date, few studies have been reported regarding the role of the cathode unit in device stability against harsh environmental conditions. Therefore, the stability of an Ag : Mg (with a higher percentage of Ag) cathode with such co-deposited EIL materials under UV-exposure needs to be investigated for use in practical applications.

**Table 1:** Comparison of properties of Ag, Mg, and Yb

Properties	Ag	Mg	Yb
Atomic weight	107.87	24.31	173.04
Atomic radius [Å]	1.65	1.45	2.22
Crystal structure	FCC	HPC	FCC
Bulk resistivity (μΩ m) at 20 °C	0.0159	0.042	0.28/0.250 (at RT)
Self-diffusion coefficient (cm^2^ s^−1^)/temperature range	6.1–6.6 × 10^−9^/903–1228 (K)	24–31 × 10^−9^/741–900 (K)	2.1 × 10^−9^/996–1076 (K)
Work function (eV)	4.26–4.74	3.66	2.60

In this study, we investigate the influence of UV radiation on the loss of emission intensity and the performance of TEOLED devices. The red, green, and blue TEOLEDs were fabricated by employing an Ag : Mg (10 : 1 wt%, 16 nm) cathode and different EILs. Exposure to UV radiation causes a loss in emission intensity and a rapid increase in operational voltage as well as shrinkage of the active pixel area over time. Here, we incorporate a co-deposited EIL, Yb : LiF, to improve the UV reliability of the fabricated TEOLEDs. Luminance decay and current density–voltage (*J*–*V*) characteristics of TEOLEDs were measured in TEOLEDs with (for 330 h) and without UV irradiation, and later transmission electron microscope (TEM)-focused ion beam (FIB)/energy dispersive X-ray spectroscopy (EDS) analysis was also performed to gain more insight into the distribution of the EIL and cathode materials. Our analysis indicates that a Yb : LiF EIL prevents the degradation of the organic/metal cathode interface by UV irradiation, inhibits pixel shrinkage, arrests luminance decay, and improves device stability.

## Experimental

*N*,*N*′-Di[4-(*N*,*N*′-diphenylamino)phenyl]-*N*,*N*′-diphenylbenzidine (DNTPD) and 1,4,5,8,9,11-hexaazatriphenylene-hexacarbonitrile (HATCN) were used as a hole injection layer (HIL), and an additional hole transport layer (HTL) from the EM Index, respectively. *N*,*N*′-Bis(naphthalen-1-yl)-*N*,*N*′-bis(phenyl)benzidine (NPB) was used as an HTL and capping layer (CPL), bis(10-hydroxybenzo[*h*]quinolinato)beryllium complex (Bebq_2_) and 4,7-diphenyl-1,10-phenanthroline (Bphen) were incorporated as a red host and electron transport layer (ETL), respectively, and were obtained from Jilin OLED Materials Tech. Iridium(iii)bis(4-methyl-2-(3,5-dimethylphenyl)quinolinato-*N*,C2′)acetylacetonate (Ir(mphmq)_2_(acac)) was synthesized using our previously reported method.^17^ Mg was purchased from Kurt J. Lesker, and Ag from Taewon Scientific. Both Mg and Ag were used to fabricate the semi-transparent cathode layer. To fabricate the OLED device, initially bare glass and strong reflective anode coated glass substrates were cleaned using sonification in acetone and isopropylalcohol (IPA) for 10 minutes each.

The cleaned substrates were finally rinsed using deionized (DI) water, followed by UV-ozone treatment for 10 minutes. All organic materials and cathode units were deposited on the pre-cleaned substrates using a vacuum evaporation technique under a pressure of 10^−7^ Torr. The deposition rate of all organic layers for HTL, EML, and ETL was about 0.5 to 0.7 Å s^−1^. The deposition rates of Yb, LiF, Mg, and Ag were 0.24 Å s^−1^, 0.24 Å s^−1^, 1.0 Å s^−1^ and 0.1 Å s^−1^, respectively. All devices were encapsulated in a glass-to-glass epoxy sealed package with a desiccant and then treated by thermal annealing at 90 °C for 60 min. In this study, the following phosphorescent red TEOLEDs were fabricated and investigated.

Device structure: Ag (100 nm)/ITO (10 nm)/DNTPD (75 nm)/HATCN (7 nm)/NPB (123 nm)/Bebq_2_: 3% Ir(mphmq)_2_(acac) (20 nm)/Bphen (40 nm)/EIL/Mg : Ag (1 : 10, 16 nm)/NPB (60 nm).

Device A: EIL – Mg : LiF (1 : 1, 2 nm).

Device B: EIL – Yb : LiF (1 : 1, 2 nm).

The current density–voltage–luminance (*J*–*V*–*L*) characteristics, and electroluminescence (EL) spectra with the CIE color coordinate of the devices were measured using a Keithley 236 and a PR-705 spectrophotometer. In order to evaluate the exact thickness and the elemental ratio, TEM (JEOL JEM 2100F)-FIB (FEI Scios), and EDS (Oxford X-Max 80T) were performed. The pixel images were measured using a Nikon microscope (ECLIPS L300N). UV irradiation on the OLED devices was performed using an Atlas Ci-5000+ model, in which the emission was generated by a xenon arc lamp with borosilicate filter (inner) + soda lime filter (outer).

## Results and discussion

### Mg : LiF (1 : 1, 2 nm) (EIL)/Ag : Mg (16 nm) cathode unit

Earlier, we showed that the optimized Ag : Mg cathode, deposited by vacuum thermal evaporation with a 10 : 1 ratio (wt%), has a sheet resistance as low as 5.2 Ω □^−1^, an average transmittance of 49.7%, a reflectance of 41.4%, and an absorbance of 8.9% over the visible spectral region (400 to 700 nm).^18^ A very clean and continuous film of Ag : Mg (10 : 1, 16 nm) cathode was observed when deposited on the Mg : LiF (1 : 1, 2 nm) film.^1^ Also, previously, NPB was reported as a good capping layer on the cathode to enhance the OLED light extraction efficiency.^18^ Therefore, in the present study, we fixed an Mg : LiF (1 : 1, 2 nm)/Ag : Mg (10 : 1, 16 nm)/NPB cathode unit to investigate the influence of UV radiation on the device performance. We used a UV spectral light intensity of 1.2 W m^−2^ at 420 nm and 35 °C temperature/50% humidity environmental conditions for irradiation. That is similar to the Global Solar Radiation data in Miami, US (Average Miami sunlight 26° south direct).^19^ The UV light was irradiated on the whole area of our fabricated red devices without any current or voltage supply.

**Fig. 1:**
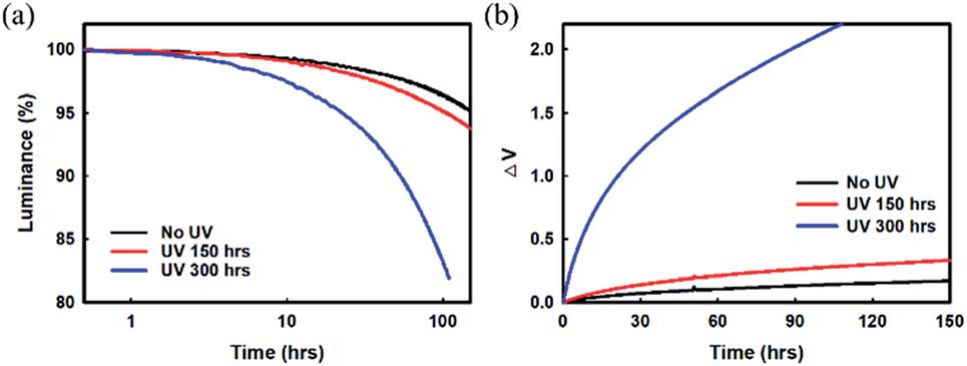
(a) Lifetime and (b) voltage variations of UV-irradiated red devices: Ag (100 nm)/ITO (10 nm)/DNTPD (75 nm)/HATCN (7 nm)/NPB (123 nm)/Bebq_2_: 3% Ir(mphmq)_2_(acac) (20 nm)/Bphen (40 nm)/Mg : LiF (1 : 1, 3 nm)/Mg : Ag (16 nm)/NPB (60 nm).

First, we measured the luminance decay and driving voltage variations over time in our red device before and after UV irradiation at 10 mA cm^−2^, as shown in Fig. 1. The lifetime and operating voltage showed almost no change in the red device that had been UV irradiated for 150 h compared with the as-fabricated device without UV exposure. However, a rapid decrease in luminance and a drastic increase in driving voltage were noticed after 8 h in the device that had been UV irradiated for 300 h. These results indicate that the device properties are significantly degraded by UV exposure.

Clearly, the decrease in luminance and the drastic increase in driving voltage over time in pre-UV irradiated devices, as displayed in the results of Fig. 1, may be the result of (i) changes/deformation of the ITO anode; degradation of the ITO/organic interface, (ii) degradation of the organic layer materials, or (iii) deformation of the metal cathode/cathode interface. The first and second reasons for degradation/deterioration are ruled out based on the results reported by earlier researchers.^20–22^ We believe that the loss of luminance and rapid increase in operational voltage may be due to some changes/deformation in the EIL/metal cathode unit. Thus, improving the stability of the EIL/Ag : Mg (10 : 1, 16 nm) metal cathode unit is very desirable to achieve a high-performance TEOLED with stable characteristics under UV exposure.

**Fig. 2:**
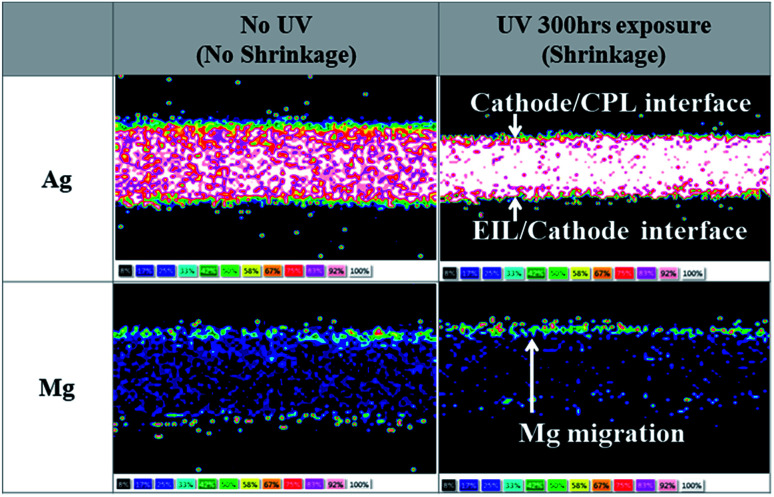
Microscopic analysis of metal component distribution by TEM/EDS in an Mg : LiF (1 : 1, 2 nm)/Ag : Mg (10 : 1, 16 nm)/NPB (60 nm) cathode unit before and after UV irradiation. A brighter area changing from a black to a white color means more atoms are present for Ag and Mg.

Therefore to understand the origin of the deterioration in device performance, we investigated the changes, if any, that occurred in an Mg : LiF (1 : 1, 2 nm)/Ag : Mg (10 : 1, 16 nm) cathode unit due to UV irradiation. It is well known that the physical distribution of each element in the co-deposited cathode layer plays a significant role in determining the performance and stability of the cathode unit. Therefore, to examine the physical distribution of each element in an Mg : LiF (1 : 1, 2 nm)/Ag : Mg (10 : 1, 16 nm)/NPB (60 nm) cathode unit, we performed the lift-out technique in FIB (Focused Ion Beams)-TEM and EDS mapping before and after UV irradiation for 300 h, as shown in Fig. 2. The lift-out FIB-TEM technique has a superior advantage in that it can directly measure the physical distribution of each element from the bulk samples without any cutting or polishing process.^23–25^ In the case of the Mg : LiF (1 : 1, 2 nm)/Ag : Mg (10 : 1, 16 nm)/NPB (60 nm) cathode structure without UV treatment, Ag and Mg atoms were dispersed uniformly throughout the entire region of the cathode. Exceptionally, some Mg atoms showed a distribution in the surface region between the EIL and the cathode, and the top of the cathode region. Mg atoms in the surface region were due to its existence in the Mg : LiF (1 : 1) EIL. The top of the cathode region is caused by the lower atomic weight of Mg than that of Ag. When the Mg and Ag were co-deposited, there was a rearrangement of atoms in the growing film and Mg with low atomic weight moved in an upward direction. In the case of UV irradiation for 300 h, Ag atoms migrated toward the center of the cathode, Mg atoms migrated toward the CPL, and no Mg atoms were observed in the EIL area after UV irradiation. The migration of Mg atoms could also be understood from their self-diffusion property, which is much higher than that of Ag.^17^ Earlier, Kai Yan *et al.* reported that metals with a smaller work function are more likely to migrate to the surface layer relative to metals with a higher work function by UV radiation (a low pressure mercury lamp with a power of 7 W and wavelength of 245 nm) due to the increase in defects in the grain boundaries.^26^ Also, Yun Cui *et al.* showed that small metal ions easily diffused into the coating from the substrate, whereas larger metal ions had more difficulty doing so because of their large atomic radii.^27^ Consequently, our subsequent investigation was focused on searching for an EIL which has excellent stability against UV irradiation and acceptable injection properties.

### Yb : LiF (1 : 1, 2 nm) (EIL)/Ag : Mg (16 nm) cathode unit

As described earlier, several bilayer metal cathodes, such as LiF/Al, alkali and alkaline earth metals, two-layer cathode units with a first layer comprising co-deposited LiF or Liq and rare earth metals, *etc.* have been employed.^10,11^ We believe that the cathode unit comprising an EIL as a co-deposited LiF and Yb metal (LiF : Yb) could become a most suitable alternative with better electron injection properties, and excellent stability against UV irradiation owing to the physical properties of Yb metal [see Table 1]. In summary, we consider that Yb metal can become a good candidate for the cathode unit, providing better electron injection properties and stability against harsh environmental conditions owing to its low self-diffusion coefficient.

**Fig. 3:**
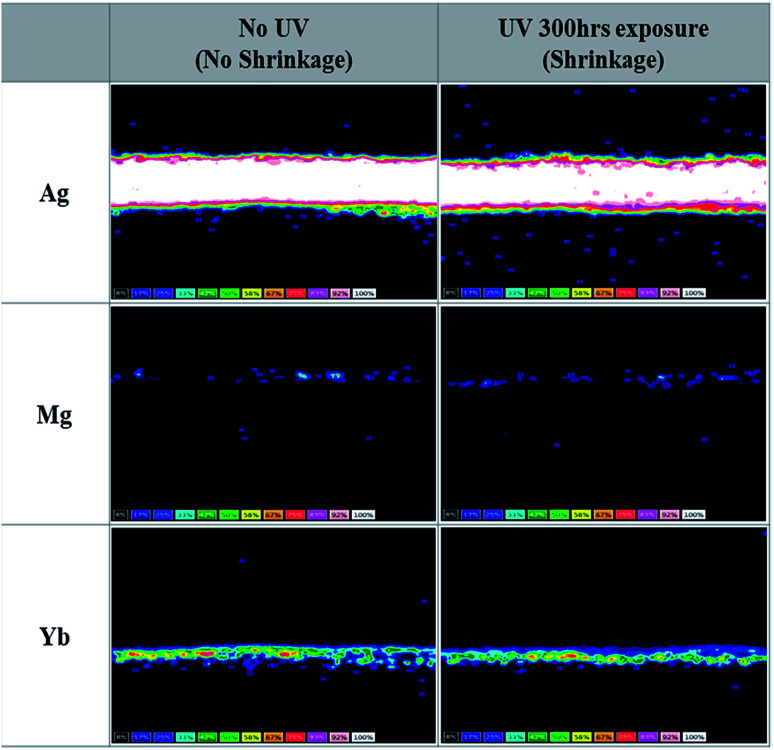
Microscopic analysis of metal component distribution by TEM/EDS in a Yb : LiF (1 : 1, 2 nm)/Ag : Mg (10 : 1, 16 nm)/NPB (60 nm) cathode unit before and after UV irradiation.

To gain more insight into the Yb : LiF (1 : 1, 2 nm)/Ag : Mg (10 : 1, 16 nm)/NPB (60 nm) cathode unit of Device B, we performed the lift-out technique in FIB-TEM and EDS mapping before and after UV irradiation for 300 h, as shown in Fig. 3. Unlike the Mg : LiF (1 : 1, 2 nm)/Ag : Mg (10 : 1, 16 nm)/NPB (60 nm) cathode unit, the results showed (i) no substantial migration of Ag atoms and they were located at the center of the cathode, (ii) Mg atoms remain unchanged with no migration toward the CPL, and (iii) no movement of Yb atoms after UV irradiation. One could also expect the movement of Yb atoms in such a cathode unit as the work function of Yb (2.60 eV) is significantly lower than that of Mg (3.66 eV) (see Table 1).^26^ However, the Yb atom has a larger atomic radius and higher atomic weight than either Mg or Ag atoms (Table 1), which inhibits the migration of atoms, as discussed by Y. Cui *et al.*^27^ Hence, through these observations, we can confirm the UV stability of the cathode unit with a Yb : LiF EIL. On comparing the results before and after UV irradiation, almost negligible changes in the cathode unit are observed in Device B. This may also contribute to the better performance of TEOLDs with a Yb : LiF (1 : 1, 2 nm)/Ag : Mg (10 : 1, 16 nm) cathode unit.

### Device characteristics

**Fig. 4:**
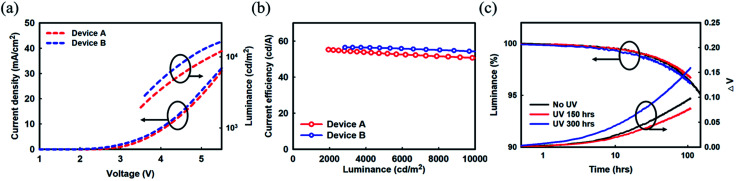
(a) Current density and luminance *versus* voltage curve and (b) current efficiency *versus* luminance characteristics. (c) Lifetime and voltage variation of Device B.

Two red phosphorescent TEOLED devices with cathode units comprising Mg : LiF (1 : 1, 2 nm)/Ag : Mg (10 : 1, 16 nm) (Device A), and Yb : LiF (1 : 1, 2 nm)/Ag : Mg (10 : 1, 16 nm) (Device B) were fabricated to investigate their electrical properties. First, the *J*–*V* and current efficiency–luminance characteristics of fabricated red phosphorescent TEOLED devices A and B are shown in Fig. 4(a) and (b), respectively. We have summarized the device characteristics in Table 2. The *J*–*V* characteristics of Device A with Mg : LiF (1 : 1, 2 nm) and Device B with Yb : LiF (1 : 1, 2 nm) were almost identical (before UV irradiation). A similar *J*–*V* tendency corroborates that both EIL could improve the electron injection property from the cathode to the electron transport layer (ETL) and the interface resistance as well. A uniform and relatively stable current efficiency of over 50 cd A^−1^ in the luminance range of 10 000 cd m^−2^ was noticed in both Devices A and B (see Fig. 4(b)). Device B showed somewhat lower current efficiency values in the measured luminance range than Device A due to the higher absorption property of Yb than that of Mg.^28^ Because of the difference in influence in the absorption property, the current efficiencies of Devices A and B were about 51 and 55 cd A^−1^ at a constant luminance of 6000 cd m^−2^. It may also be noted that Devices A and B exhibited a very low efficiency roll-off, from the maximum efficiency value to that of 10 000 cd m^2^; their current efficiency roll-offs were only 5.35% (56 to 53 cd A^−1^) for Device A and 9.25% (54 to 49 cd A^−1^) for Device B. Although the efficiency roll-off values were less than 10% from maximum efficiency to 10 000 cd m^−2^, the difference in efficiency roll-off between Devices A and B was significant. It is argued that the different micro-cavity effects due to the difference in absorption characteristics of cathode units contributes to their different efficiency roll-off behaviors. In summary, almost uniform current efficiency with luminance in fabricated TEOLEDs helps to ensure a stable and efficient OLED for practical applications.

**Table 2:** Summarized characteristics of Devices A and B

Device	UV exposure	Voltage (V)	Efficiency (cd A^−1^)	Peak (nm)	FWHM (nm)	Lifetime (LT_95_)
Device A	0 h	4.5^a^	55.2^b^	610^a^	29^a^	155
	300 h	4.9	—	610	27	22
Device B	0 h	4.3	56.5	608	30	155
	300 h	4.3	—	608	30	150

a At 3000 units.

b At 10 mA cm^−2^.

Later, several red phosphorescent TEOLEDs with device structures A and B were fabricated for UV radiation experiments (Fig. 1(a), (b) and 4(c)). The fabricated devices were exposed to UV irradiation by a xenon arc lamp at 420 nm, and a temperature of 35 °C/50% humidity environmental conditions for varying time periods. The driving voltage of these Devices A and B after UV irradiation (300 h as a representative time duration), accompanied by the driving voltage results of un-irradiated devices for a better comparison, are summarized in Table 2. In Device A with an Mg : LiF (1 : 1, 2 nm) EIL, a remarkable increase in the voltage variation curve compared to the un-irradiated device was noticed, as can be seen in Fig. 1(b). This increase in voltage indicates a deterioration in electron injection from the cathode to the ETL, as observed in the Mg diffusion at the cathode interface. On the other hand, no such changes were observed in the voltage variation curve of irradiated and un-irradiated Device B with a Yb : LiF (1 : 1, 2 nm) EIL, indicating that there is no change in electron injection behavior with UV irradiation. The interface between Yb : LiF (1 : 1, 2 nm) and the metal cathode seems to be intact; hence there is a similar interface resistance regardless of UV irradiation.

Additionally, EL spectra of fabricated Devices A and B were almost the same, as noted in Table 2. Device A shows a peak wavelength at 610 nm and a full width at half maximum (FWHM) of 29 nm. However, the spectrum of Device A changed when it was exposed to UV irradiation for 300 h. After UV irradiation, the spectrum narrowed to a 27 nm FWHM from 29 nm with the same peak wavelength, which can be attributed to an increase in the micro-cavity effect by some morphological changes in the cathode. Similar to *J*–*V* characteristics as displaced in Fig. 1(a), the changes in the EIL/cathode interface also affect the optical characteristic in Device A. There are excessive Ag atoms migrating toward the center of the cathode after UV irradiation, which then increase the micro-cavity effect (see results of Fig. 2). Whereas Device B displayed the same spectrum regardless of UV irradiation (peak wavelength of 608 nm and FWHM of 30 nm). No changes in the EL spectrum of Device B could be attributed to the stable interface property of the EIL/cathode unit. Earlier, Yun *et al.* showed that the composite layer of LiF : Yb is appropriate for injecting electrons from a cathode and for transport to an EML, and has excellent adhesion characteristics for Ag film.^12^ Device B with a Yb : LiF EIL is robust against harsh environmental conditions, such as UV irradiation, compared to Device A with Mg : LiF EIL. Clearly, the underlying Yb : LiF (1 : 1, 2 nm) layer of the Ag : Mg (10 : 1, 16 nm) cathode seems to play a vital role in slowing the decay in luminance.

Next, to acquire more insight into the effect of UV irradiation on the performance of TEOLEDs, the initial luminance before UV exposure was taken as a reference and the relative luminance (%) variation with duration of UV exposure time was measured. 95% of the reference luminance value is taken as a standard for commercial applications. Device A with an Mg : LiF (1 : 1, 2 nm) EIL lost 5% of its luminance and reached the 95% relative luminance value in 155 h before UV irradiation. After UV irradiation for 300 h, it reached the 95% relative luminance value in 22 h. A decrease in lifetime means that serious degradation has occurred in Device A due to UV exposure. On the other hand, Device B with a Yb : LiF (1 : 1, 2 nm) EIL reached the 95% relative luminance value in 155 h and this value is similar to that of Device A without UV exposure. After UV irradiation, it remained 150 h at the 95% relative luminance value and there was almost no change regardless of UV exposure. We can expect that Device B which has a Yb : LiF EIL showed stable driving characteristics under UV exposure conditions. Likewise, this tendency of the measured results matches the *J*–*V* curves and EL spectra discussed earlier. As a result, the drop in the luminance with UV irradiation was improved by applying Yb to the EIL in OLED devices. Clearly, the underlying Yb : LiF (1 : 1, 2 nm) layer of the Ag : Mg (10 : 1, 16 nm) cathode seems to play a vital role in slowing the decay in luminance.

Undoubtedly, UV irradiation affects the variation in driving voltage (see Fig. 1 and 4). The electron injection property of an Ag : Mg cathode had deteriorated after UV exposure by a significant amount. The inferior behavior of the increased driving voltage is due to the degradation in the interface between the EIL and the metal cathode, and the increase in interface resistance, thereby reducing the electron injection property from the cathode to the ETL. When UV or thermal energy is applied to such a cathode film, the thin film grows to form larger islands that become more stable, leading to cathode deformation. Such a deformed cathode interferes with the injection of electrons, thereby reducing the brightness of the pixels. In contrast, no such change in Device B with a Yb : LiF (1 : 1, 2 nm)/Ag : Mg (10 : 1, 16 nm) cathode unit was noticed. Furthermore, the *J*–*V* characteristic behavior is negligibly influenced after UV exposure, as shown in Fig. 4(c). The results show that the EIL/cathode interface remains unaffected by UV exposure. The presence of underlying Yb in the Yb : LiF (1 : 1, 2 nm) EIL seems to be contributing to the stability of the Ag : Mg (10 : 1, 16 nm) cathode. In fact, we were expecting such a result in a fabricated device with a Yb : LiF EIL. The surface energies of LiF, Yb, and Mg, are about 0.48, 0.45 to 0.46, and 0.64 J m^−2^, respectively.

Although the surface energies of these materials are much lower than the 1.2 J m^−2^ of Ag, the small thickness of these films (about 1 to 2 nm) acts as a wetting layer to form an amorphous smooth Ag film during the deposition process. However, the presence of Yb in the EIL provides more stability, and no change in electron injection behavior against external factors such as UV radiation as compared to Mg. As a result, a composite layer of LiF : Yb is appropriate for injecting electrons from a cathode and for transporting electrons to an EML as well as having excellent adhesion characteristics for Ag film.^29^

### RGB TEOLEDs pixel shrinkage

Finally, to evaluate the performance of a Yb : LiF (1 : 1, 2 nm) EIL in arresting pixel shrinkage, again RGB TEOLEDs with a Yb : LiF (1 : 1, 2 nm)/Ag : Mg (10 : 1, 16 nm) cathode unit were implemented, and the results were compared with devices with an Mg : LiF (1 : 1, 2 nm)/Ag : Mg (10 : 1, 16 nm) cathode unit. Each color of TEOLED device was fabricated with the following structures:

Red device: Ag (100 nm)/ITO (10 nm)/DNTPD (75 nm)/HATCN (7 nm)/NPB (123 nm)/Bebq_2_: 3% Ir(mphmq)_2_(acac) (20 nm)/Bphen (40 nm)/EIL/Mg : Ag (10 : 1 wt%, 16 nm)/NPB (60 nm).

Green device: Ag (100 nm)/ITO (10 nm)/DNTPD (75 nm)/HATCN (7 nm)/NPB (90 nm)/Bepp_2_: 3% Ir(ppy)_2_(acac) (20 nm)/Bphen (40 nm)/EIL/Mg : Ag (10 : 1 wt%, 16 nm)/NPB (60 nm).

Blue device: Ag (100 nm)/ITO (10 nm)/DNTPD (75 nm)/HATCN (7 nm)/NPB (58 nm)/MADN: 3% BCzVBi (20 nm)/Bphen (25 nm)/EIL/Mg : Ag (10 : 1 wt%, 16 nm)/NPB (60 nm).

**Fig. 5:**
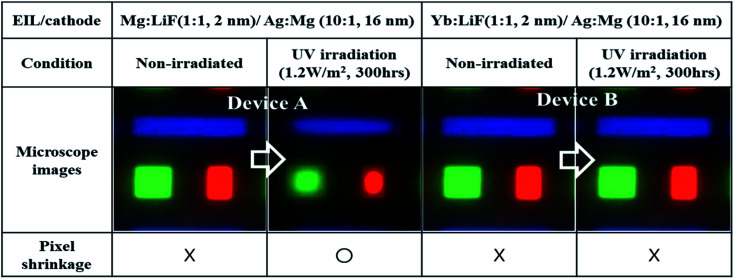
Microscopic images of the active pixel area of devices before and after UV irradiation. Devices are composed of an Mg : LiF (1 : 1, 2 nm)/Ag : Mg (10 : 1, 16 nm) cathode unit and a Yb : LiF (1 : 1, 2 nm)/Ag : Mg (10 : 1, 16 nm) cathode unit.

RGB pixel images of the fabricated devices were measured using a Nikon microscope (ECLIPS L300N), as shown in Fig. 5.

A significant reduction in the emitting pixel area was noticed in RGB devices with Mg : LiF (EIL) after UV irradiation for 300 h. However, Yb : LiF based RGB-TEOLEDs showed an almost identical pixel area to the as-fabricated device after UV irradiation for 300 h. The loss in active area of Mg : LiF based TEOLEDs can be attributed to changes in the composition of the metal cathode unit. The undesirable appearance of dark spots and/or pixel shrinkage occurs due to the bank taper in the border region of the pixels. This area/region is relatively thin due to the slope. Even if the performance of the device has decreased over the whole area, degradation in the border region has occurred more quickly than in the central region. Additionally, the shrinkage of an active pixel area may be far more significant for small pixel sizes and/or micro-displays. In OLED display devices, such pixel shrinkage is a major concern to industries, academics, and users. Usually, the reduced pixel region results in non-uniform brightness, a gradient in emission intensity, and so on.

## Conclusions

The loss of luminescence intensity, rapid increase in operational voltage over time and undesirable appearance of dark spots and/or pixel shrinkage after UV irradiation limit the reliability of devices against operation in harsh environmental conditions, and are also the major cause of product failure. We investigated the stability of red phosphorescent TEOLEDs under UV irradiation/solar radiation by employing a Yb : LiF EIL underneath the Ag : Mg cathode in the device. Highly stable variation in luminescence intensity over time was noticed in a previously UV-irradiated (for 300 h) red TEOLED device with a Yb : LiF (1 : 1, 2 nm)/Ag : Mg (10 : 1, 16 nm)/NPB (60 nm) cathode unit. The operational voltage had also not increased significantly. Moreover, no change in the physical distribution of each constituent in a co-deposited metal cathode Yb : LiF (1 : 1, 2 nm)/Ag : Mg (10 : 1, 16 nm)/NPB (60 nm) cathode unit was observed, as this layer plays a significant role in determining the performance and stability of the cathode unit. Furthermore, a uniform and relatively stable current efficiency of over 50 cd A^−1^ in the luminance range 10 000 cd m^−2^ was noticed in a red phosphorescent device with a Yb : LiF (1 : 1, 2 nm)/Ag : Mg (10 : 1, 16 nm) cathode unit before UV irradiation. A current efficiency of about 51 cd A^−1^ at a given constant luminance of 6000 cd m^−2^ and a low current efficiency roll-off of 9.25% (54 to 49 cd A^−1^) were observed in this device. In addition, concerns about pixel shrinkage and/or dark spot formation were addressed by having a more robust Yb : LiF EIL. Our TEOLED device with a Yb : LiF (1 : 1, 2 nm) EIL decelerates the aging effect, arrests pixel shrinkage, and improves device stability and performance against UV exposure, paving the way for applications in harsh environmental conditions, automobiles, and space applications.

## Conflicts of interest

There are no conflicts to declare.

## Supplementary Material
